# Clinical use and predictors of outcome in venoarterial extracorporeal membrane (VA ECMO): insights from VERGE (VA ECMO Registry of Germany)

**DOI:** 10.1007/s00392-025-02650-3

**Published:** 2025-04-22

**Authors:** Tobias Wengenmayer, Dawid L. Staudacher, Alois Philipp, Eike Tigges, Angela Dettling, Hendrik Busse, Marc Kriege, Jan-Sören Padberg, Ingo Voigt, Clemens Scherer, Tobias Graf, Dominik Scharpf, Peter Noack, Simone Britsch, Guido Michels, the VERGE Study Group, Dirk Lunz, Thomas Müller, Adrian Springer, Piotr Foszcz, Benedikt Schrage, Stefan Kluge, Alexander Supady, Sven Maier, Aiman Alken, Ingo Sagoschen, Felix Rosenow, Jan Sackarnd, Stefan Leinen, Felix Michelson, Jan-Philipp Simon, Sven Siemonsen, Sebastian Becker, Sverrir Möller, Marcus Hennersdorf, Sebastian Münz, Simone Britsch, Simon Lindner

**Affiliations:** 1 Interdisciplinary Medical Intensive Care (IMIT), Faculty of Medicine and Medical Center, University of Freiburg, Freiburg, Germany; 2 Department of Cardiothoracic Surgery, University Medical Center Regensburg, Regensburg, Germany; 3 Asklepios Clinic St. Georg, Cardiology and Critical Care, Hamburg, Germany; 4 DZHK—Deutsches Zentrum Für Herz-Kreislauf-Forschung, Hamburg, Germany; 5 University Medical Center Hamburg-Eppendorf, Hamburg, Germany; 6 Heart and Vascular Center Bad Bevensen, Bad Bevensen, Germany; 7 Department of Anesthesiology, University Medical Center, Johannes Gutenberg-University, Mainz, Germany; 8 Department for Cardiology I: Coronary and Periphereal Vascular Disease, Heart Failure, University Hospital Münster, Münster, Germany; 9 Department of Acute and Emergency Medicine, Elisabeth-Hospital Essen, Essen, Germany; 10 Department of Medicine I, LMU University Hospital, LMU Munich, Munich, Germany; 11 University Heart Center Lübeck, Lübeck, Germany; 12 SLK-Kliniken Heilbronn GmbH, Klinikum Am Gesundbrunnen, Medical Department I, Heilbronn, Germany; 13 Department of Cardiology, Intensive Care Medicine, Klinikum Nürnberg, Paracelsus Medical University, Nuremberg, Germany; 14 Centre for Acute Cardiovascular Medicine Mannheim (DZKAM), Department of Cardiology, Angiology, Haemostaseology and Medical Intensive Care, University Medical Centre Mannheim, Medical Faculty Mannheim, Heidelberg University, Mannheim, Germany; 15 German Centre for Cardiovascular Research (DZHK), Mannheim, Germany; 16 European Centre for Angioscience (ECAS), Medical Faculty Mannheim, Heidelberg University, Mannheim, Germany; 17 Department of Emergency Medicine, Hospital of the Barmherzige Brüder, Trier, Germany

**Keywords:** Extracorporeal membrane oxygenation (ECMO), Shock, Extracorporeal cardiopulmonary resuscitation (ECPR), Outcome, Survival

## Abstract

The VA ECMO Registry of Germany (VERGE, http://va-ecmo-register.de/) is a prospective, multicenter, investigator-driven registry of Venoarterial Extracorporeal Membrane Oxygenation (VA ECMO) all-comers, free from industrial support. VERGE is Germany’s first multicenter registry to systematically gather and analyze data from various centers on the clinical use of VA ECMO. This first report compromises data from 581 VA ECMO patients from 2022. Median age was 60 years, hospital survival was 42% and 25% were female. The leading indication for VA ECMO was extracorporeal cardiopulmonary resuscitation (ECPR) followed by VA ECMO in shock (48.9 and 34.9%, respectively). Hospital survival of ECPR was significantly worse compared to shock (28 and 55%, respectively, *p* < 0.001). Age, pH, and lactate before cannulation all significantly correlated independently with hospital survival (*p* < 0.001). In VERGE, no patients with pH below 6.7 or lactate above 25 mmol/l survived.

**Supplementary Information:**

The online version contains supplementary material available at 10.1007/s00392-025-02650-3.

## Introduction

Venoarterial Extracorporeal Membrane Oxygenation (VA ECMO) represents a critical intervention in cardiovascular support, particularly for patients experiencing acute cardiac failure and cardiogenic shock [1, 2]. Although no large prospective randomized trials yet demonstrated the efficacy of VA ECMO in reducing mortality, the number of VA ECMO utilizations is growing [3]. In response to the growing use of such highly invasive procedures, a national registry of VA ECMO all-comers has been founded.

The aims of this report are (i) to give an overview of the usage of VA ECMO in Germany in 2022 and (ii) to correlate of the primary outcome with predictors of outcome in VA ECMO.

## VERGE (VA ECMO Registry of Germany)

VERGE (http://va-ecmo-register.de/) is the inaugural registry in Germany dedicated to systematic collection and analysis VA ECMO clinical data. This prospective, multicenter, investigator-led registry includes all VA ECMO cases and operates without industrial funding. This registry is endorsed by the German Society for Cardiology—Cardiovascular Research (DGK) and the German Society for Thoracic and Cardiovascular Surgery (DGTHG) and is aimed at all ECMO therapy users. The registry is open to all professional societies and explicitly invites and encourages all professional societies involved in ECMO therapy to support and participate. There is a secondary option to include data retrospectively. Reports from VERGE aim to provide a foundational understanding of the clinical practice patterns, patient outcomes, and potential areas for quality improvement within the German healthcare system. Prospectively, data pool will continue to grow, and an annual report will be created from the aggregated data. Each participating center will receive a center-specific report upon completion of the national report to enable benchmarking. This approach will facilitate continuous improvement and allow each center to compare its performance with the national standards and other centers, fostering a collaborative environment aimed at enhancing overall patient outcomes.

## Methods

In VERGE, anonymized data were collected from all participating centers in Germany and managed using REDCap (Research Electronic Data Capture) electronic data capture tools hosted at the University of Freiburg, Germany. REDCap is a secure, web-based software platform designed to support data capture for research studies [4, 5].

In VERGE, patient data categorization is mandatory. Each patient must be classified into one of the following categories based on their indication for treatment: ECPR, defined as venoarterial (VA) ECMO in patients without a stable return of spontaneous circulation (ROSC); VA ECMO for shock, applicable to all types of shock; peri-procedural VA ECMO, used as backup or to facilitate a procedure; or post-cardiotomy VA ECMO, implemented in the context of post-cardiotomy surgery. Only one category could be assigned to each patient.

We included all VA ECMO runs that began on or after January 1, 2022 to December 31, 2022 and were entered in the database by April 15, 2024. REDCap was configured to allow participating centers to see their own data only. Since this registry only comprises anonymized data presented aggregated and serves quality assurance purposes, no consent from patients was required as discussed with the ethics committee of Freiburg. Additionally, approval from regional ethics committees for the submission of data was deemed unnecessary. Data presented derive from 14 German centers, specifically.

- University Hospital Regensburg, Germany
- Asklepios Clinic St. Georg, Cardiology and Critical Care, Hamburg, Germany
- University Medical Center Hamburg-Eppendorf, Hamburg, Germany
- Interdisciplinary Medical Intensive Care (IMIT), Faculty of Medicine and Medical Center, University of Freiburg, Germany
- Heart and Vascular Center Bad Bevensen, Bad Bevensen, Germany
- Department of Anesthesiology, University Medical Center, Johannes Gutenberg-University Mainz, Germany
- Department for Cardiology I: Coronary and Periphereal Vascular Disease, Heart Failure, University Hospital Münster, Germany
- Department of Acute and Emergency Medicine, Elisabeth-Hospital Essen, Essen, Germany
- Hospital of the Barmherzige Brüder, Trier, Germany
- Department of Medicine I, LMU University Hospital, LMU Munich, Germany
- University Heart Center Lübeck, Germany
- SLK-Kliniken Heilbronn GmbH, Klinikum Am Gesundbrunnen, Medical Department I, Heilbronn, Germany
- Department of Cardiology, Intensive Care Medicine, Klinikum Nürnberg, Paracelsus Medical University, Nuremberg, Germany
- Medical Faculty Mannheim, Heidelberg University, Mannheim, Germany

### Predictors

As for this research, only indication subgroups with at least 100 entries were further differentiated. Therefore, peri-procedural or post-cardiotomy patients were not investigated as separate subgroups in the current evaluation of the registry. The predictors of hospital survival were pre-defined including age and the biomarkers pH and lactate. The primary endpoint of this research was hospital survival.

### Statistical analysis

Data are presented descriptively as counts and percentages, as well as medians and interquartile ranges (IQR) stratified by group or level. Missing values were excluded before calculating descriptive statistics (unless otherwise noted), and counts of missing values are provided, where applicable. Analysis was conducted in R Statistical Software Version 4.3.2 [6] and figures were produced using the package ggplot2 [7]. Differences between the groups were calculated using Kruskal–Wallis rank sum test, Wilcoxon rank sum test, Pearson’s chi-squared test or Fisher’s exact test, as applicable. A Pearson correlation analysis using the cor.test function in R and a binomial regression model using the glm function in R was performed to analyze the relationship between various pre-ECMO values and ICU survival. A *p* value < 0.05 was considered statistically significant.

## Results

**Fig. 1:**
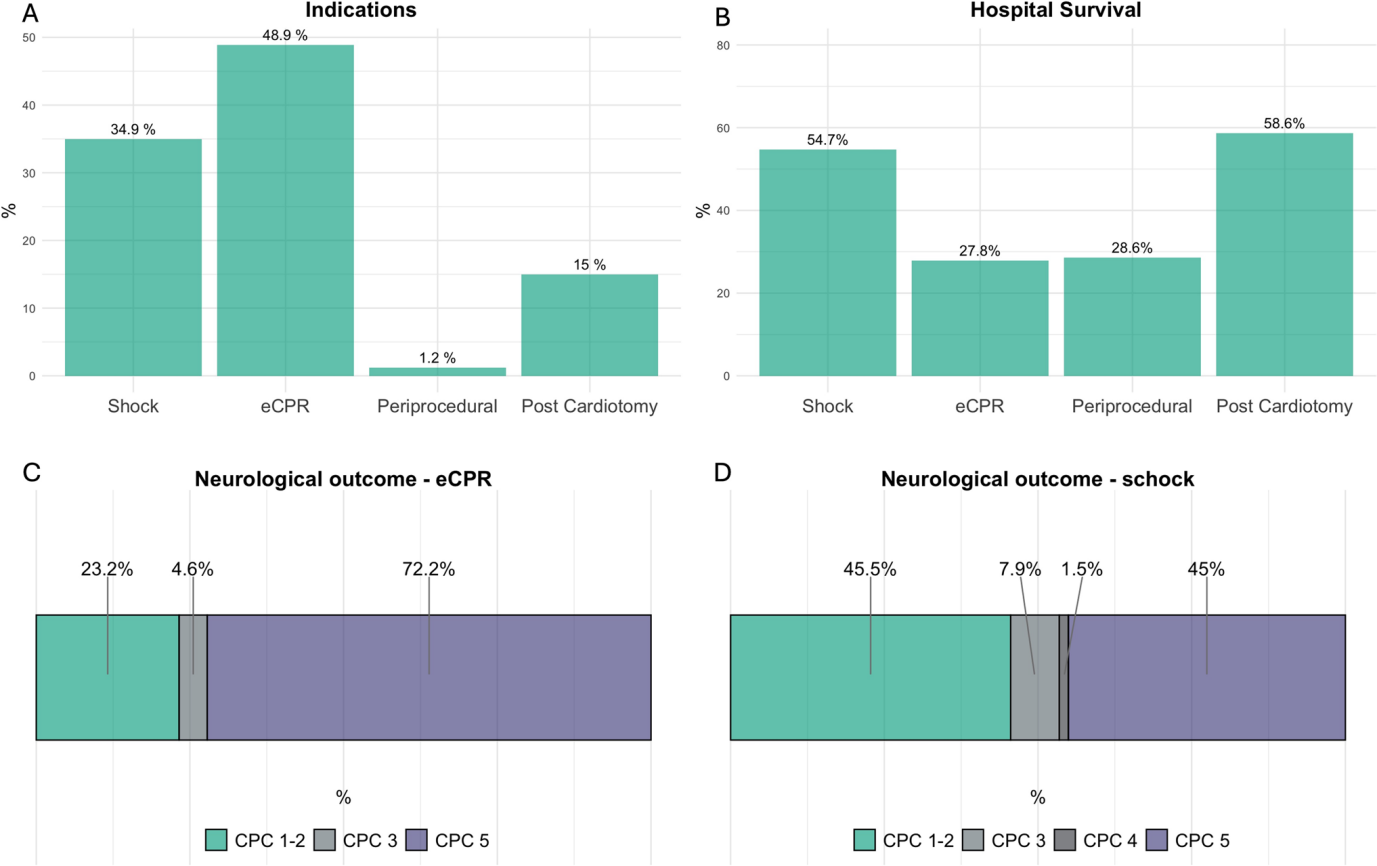
Indication and outcome of patients in VERGE. (**A**) shows indication for VA ECMO. Most patients are included either for shock or ECPR. (**B**) draws the survival according to the indication for VA ECMO. Survival is better in shock and post-cardiotomy than ECPR. The neurological outcome according to the CPC is displayed for ECPR (**C**) and shock (**D**). Most surviving patients are discharged with a favorable outcome (CPC 1–2) as shown in green

For 2022, a total of 581 patients from 14 centers were included in the VA ECMO registry of Germany (VERGE). The indications for VA ECMO support were pre-categorized into four subtypes: shock, ECPR, peri-procedural, and post-cardiotomy. The predominant indication was VA ECMO for ECPR with 48.9% and only 1% for peri-procedural support. Hospital survival was as follows: 28.2% for eCPR, 55.2% for shock, 28.6% for peri-procedural, and 59.8% for post-cardiotomy. Survivors of ECPR and VA ECMO for shock exhibited a dichotomous neurological outcome at the time of hospital discharge, characterized by either good neurological outcome (defined as Cerebral Performance Category [CPC] 1 or 2) or death (CPC 5). Among ECPR patients, 4.6% were discharged with a severe neurological impairment (CPC 3–4), opposed to 9.4% patients with VA ECMO for shock (*p* = 0.049), see Fig. 1 and Table 1.

**Table 1:** Patient characteristics in VERGE

Variable	*N*	All patients, *N* = 581	Other, *N* = 94	Shock, *N* = 203		ECPR, *N* = 284	*p* value^$^
Age, median (IQR)	581	61 (52, 69)	66 (58, 72)	61 (53, 68)		60 (51, 68)	< 0.001
Male gender, *n* (%)	581	436 (75%)	70 (74%)	145 (71%)		221 (78%)	0.273
Type of shock, * n* (%)	231						< 0.001
Cardiogenic		176 (76%)	9 (35%)	167 (82%)		0 (0, 0)	
Non-cardiogenic		55 (24%)	17 (65%)	36 (18%)		0 (0, 0)	
Witnessed arrest, * n* (%)	296	272 (92%)	5 (100%)	9 (90%)		258 (92%)	0.728
No-flow duration (minutes), median (IQR)	216	0 (0, 2)	0 (0, 0)	0 (0, 0)		0 (0, 2)	0.125
Bystander CPR, * n* (%)	271	199 (73%)	5 (100%)	8 (89%)		186 (72%)	0.330
Mechanical CPR, * n* (%)	281	190 (68%)	0 (0%)	10 (83%)		180 (68%)	0.003
Low-flow duration (minutes), median (IQR)	275	45 (28, 70)	12 (0, 14)	20 (16, 31)		47 (30, 70)	< 0.001
Lactate pre-implant (mmol/l), median (IQR)	527	9.3 (5.5, 14.0)	8.6 (4.8, 12.0)	7.2 (3.7, 11.2)		11.8 (8.0, 15.5)	< 0.001
pH pre-implant, median (IQR)	518	7.14 (7.00, 7.27)	7.20 (7.11, 7.29)	7.23 (7.10, 7.35)		7.04 (6.90, 7.16)	< 0.001
Venting, * n* (%)	581	135 (23%)	24 (26%)	52 (26%)		59 (21%)	0.389
Time on ICU, median (IQR)	526	11 (3, 21)	16 (11, 24)	15 (6, 25)		4 (1, 16)	< 0.001
Successful ECMO weaning, * n* (%)	580	299 (52%)	63 (67%)	129 (64%)	107 (38%)		< 0.001
30-day survival, * n* (%)	576	245 (43%)	54 (57%)	104 (52%)	87 (31%)		< 0.001
Hospital survival, * n* (%)	580	243 (42%)	54 (57%)	112 (55%)	80 (28%)		< 0.001
CPC at discharge, * n* (%)	580						< 0.001
CPC 1–2		201 (35%)	43 (46%)	92 (46%)	66 (23%)		
CPC 3–4		42 (7.2%)	10 (11%)	19 (9.4%)	13 (4.6%)		
CPC 5		337 (58%)	41 (44%)	91 (45%)	205 (72%)		

Patient characteristics of all patients included in VERGE. $Significance is calculated between the subgroups given in columns 4–6. The column '*N*' denotes the overall sample size with available values for each variable, irrespective of group; percentages reflect solely the observed data without any imputation

*CPC* cerebral performance category, *CPR* cardiopulmonary resuscitation, *ECMO* extracorporeal membrane oxygenation, *ECPR* extracorporeal cardiopulmonary resuscitation, *ICU* intensive care unit, *IHCA* in-hospital cardiac arrest, *IQR* interquartile range, *OHCA* out-of-hospital cardiac arrest

**Fig. 2:**
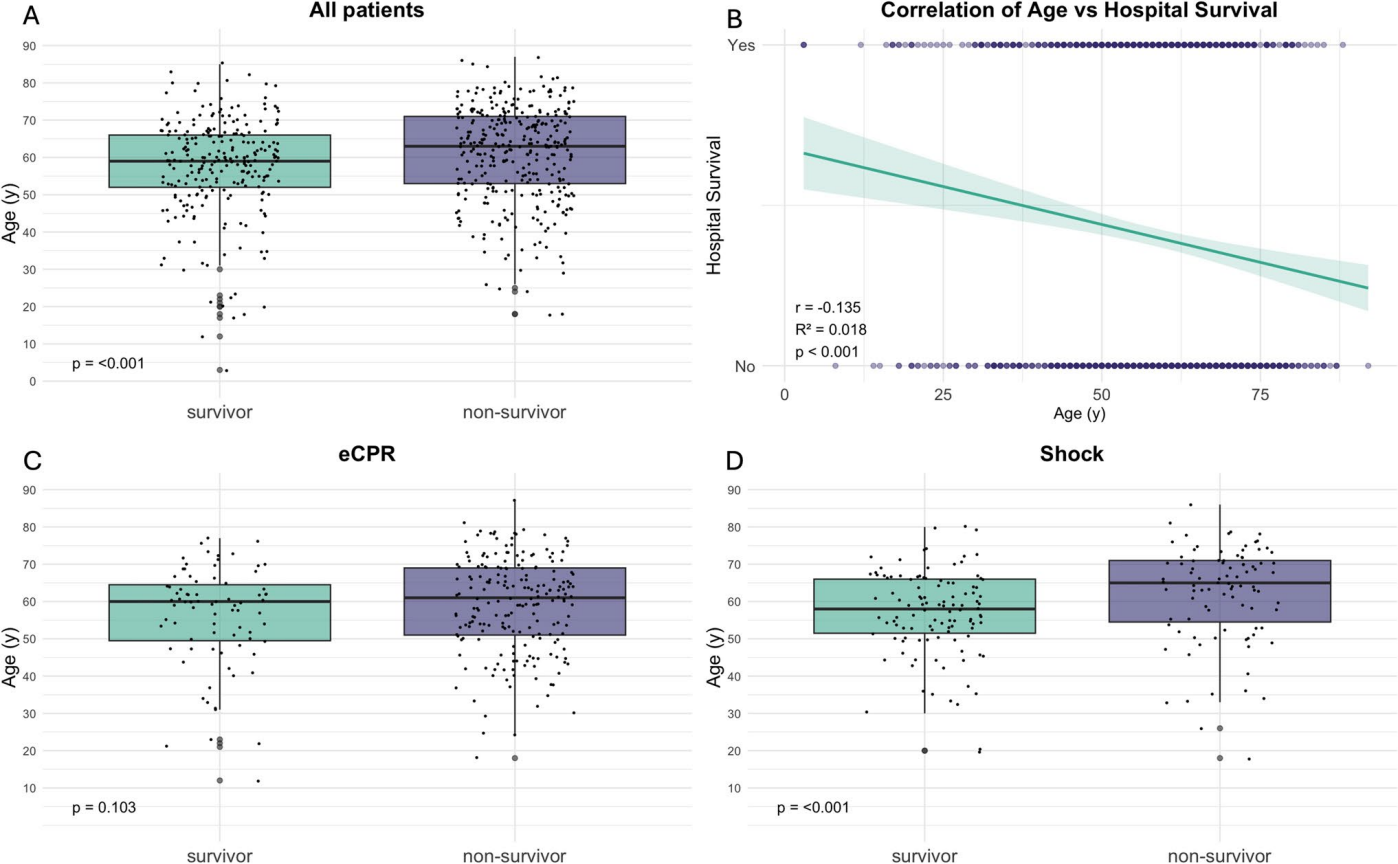
Age and outcome after VA ECMO. (**A**) shows the age at cannulation for VA ECMO survivors and non-survivors. Survivors were significantly younger. (**B**) shows a correlation of age at cannulation and survival. There was a significant correlation with worse prognosis in older patients. Age of survivors and non-survivors is shown for the two main subgroups ECPR (**C**) and shock (**D**). Survivors were younger in both groups, the difference was statistically significant only in shock patients (*p* = 0.103 and *p* < 0.001, respectively)

The median ages for patients receiving VA ECMO support in VERGE were 61 years, similar in shock and ECPR (61 and 60 years, respectively), see Table 1. Survivors were significantly younger than non-survivors (*p* < 0.001), which was consistent only in the shock subgroup, see Fig. 2A, C, D. The median age for survivors was 60 (ECPR) and 59 years (shock), compared to 61.0 (ECPR) and 65.0 years (shock) for non-survivors (*p* = 0.103 and *p* < 0.001). Pearson correlation analysis revealed a significant but weak negative correlation between age and hospital survival (*p* < 0.001). A logistic regression analysis showed that each additional year of age was associated with a 1.97% decrease in the odds of hospital survival (OR = 0.980, *p* < 0.001) in all patients, see Supplemental Fig. 1.

**Fig. 3:**
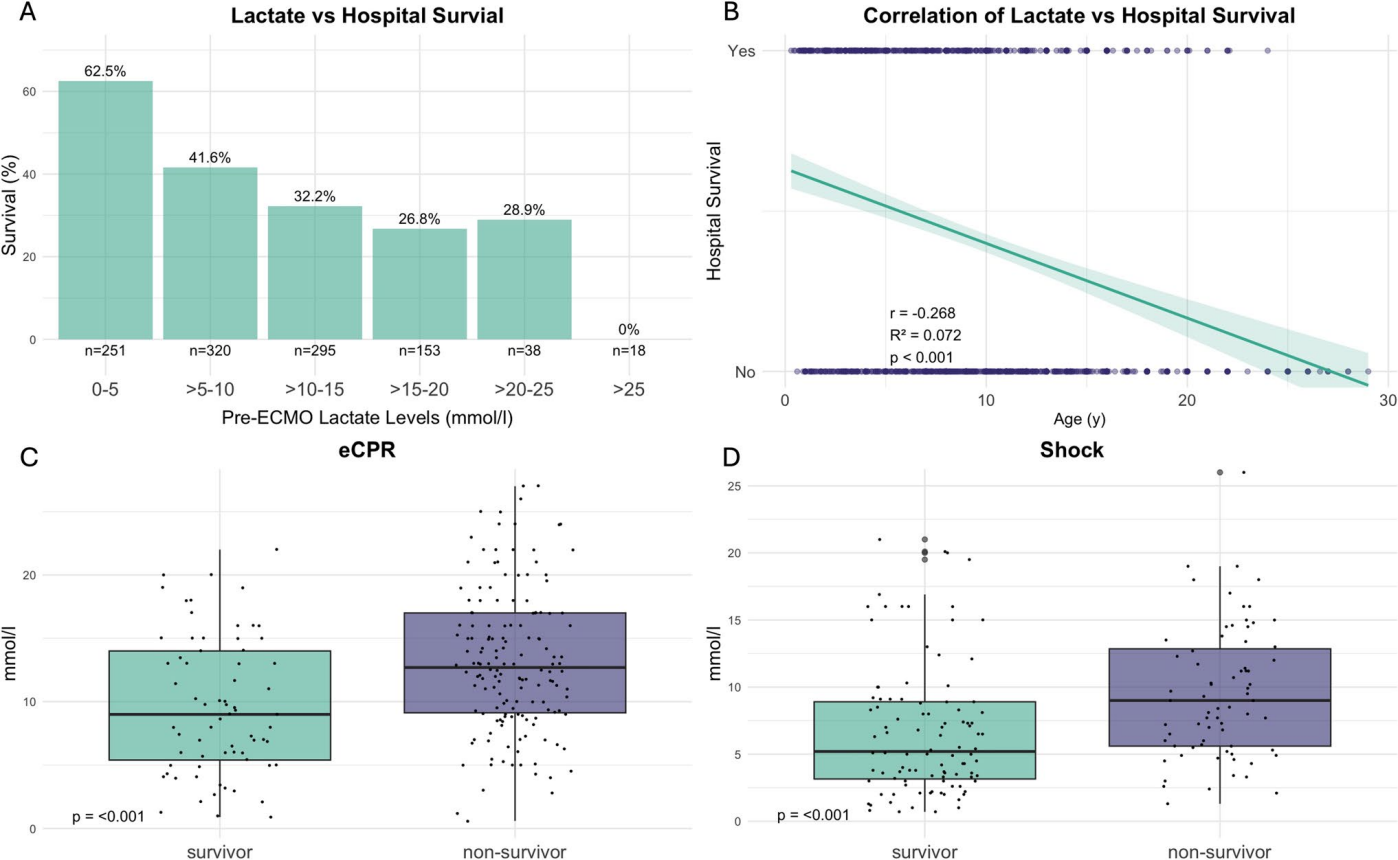
Lactate and outcome after VA ECMO. (**A**) shows hospital survival stratified by lactate before cannulation for VA ECMO. No patient survived with a lactate above 25 mmol/l. (**B**) Logistic regression of lactate before cannulation and survival. Survival approaches zero asymptotically with increasing lactate levels. Lactate before cannulation in survivors and non-survivors is shown for the two main subgroups ECPR (**C**) and shock (**D**). Survivors had significantly lower lactate values in both subgroups (both *p* = 0.001)

Lactate levels before cannulation (mmol/L) showed a significant difference between survivors and non-survivors. The median lactate level for survivors was 7.3, compared to 11.1 mmol/L for non-survivors (*p* < 0.001), see Supplemental Fig. 2. Logistic regression analysis indicated a strong and significant influence of lactate levels on survival probability (Fig. 3A, *p* < 0.001). For shock patients, lactate levels prior to implantation were significantly different between survivors and non-survivors. The median lactate level was 5.3 mmol/L for survivors compared to 9.0 mmol/L for non-survivors (*p* < 0.001). Similar results were detected in ECPR patients. The median lactate level was 9.0 and 12.4 mmol/L for survivors and non-survivors, respectively, *p* < 0.001).

**Fig. 4:**
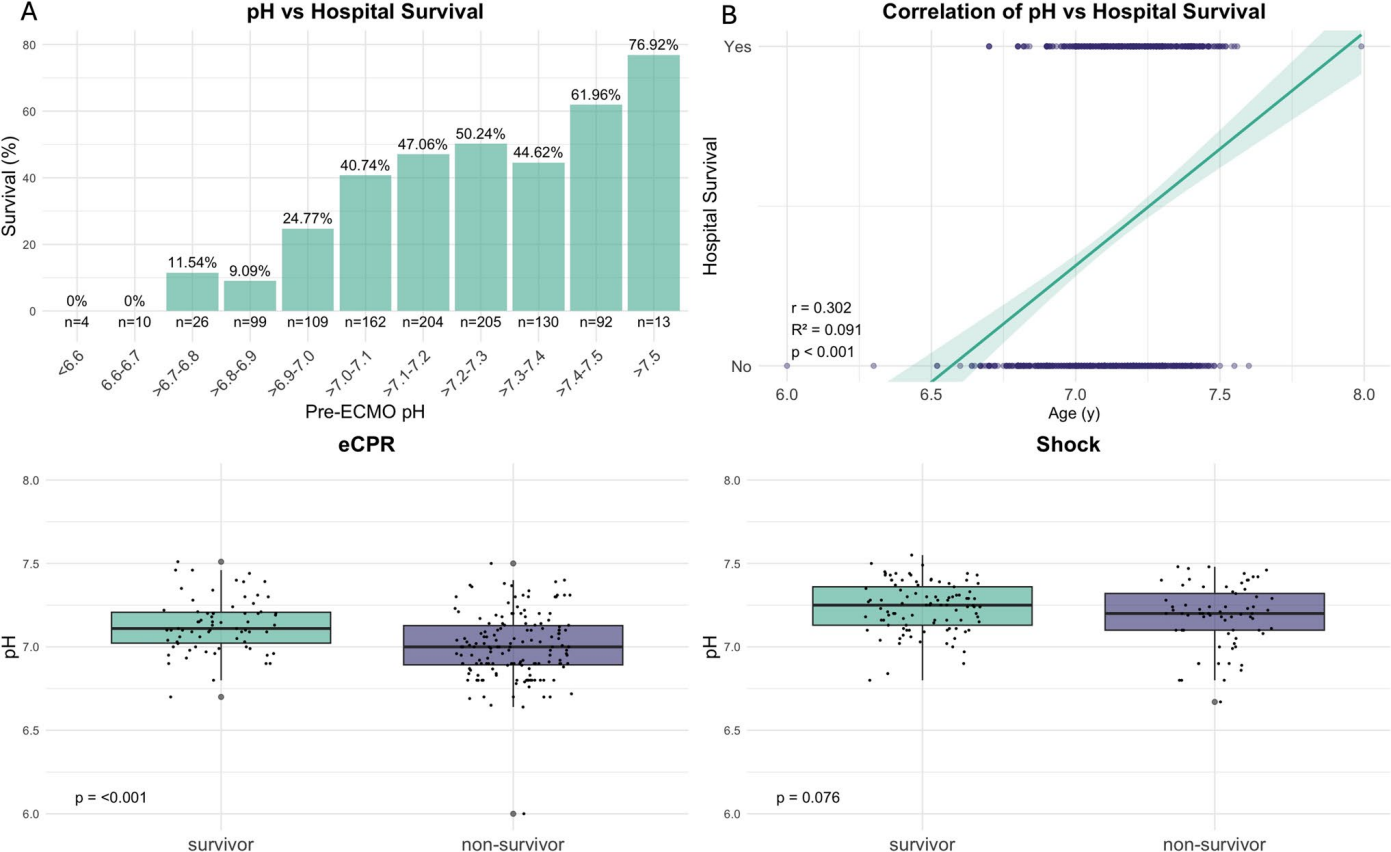
pH and outcome after VA ECMO. (**A**) shows hospital survival stratified by pH before cannulation for VA ECMO. No patient survived with a pH below 6.7. (**B**) Logistic regression of lactate before cannulation and survival. Survival probability shows a sigmoidal shape asymptotically approaching zero survival at low pH levels and 100% survival at high pH levels. pH before cannulation in survivors and non-survivors is shown for the two main subgroups ECPR (**C**) and shock (**D**). Survivors again had significantly higher pH values in the ECPR subgroup (*p* < 0.001) but not in shock patients (*p* = 0.2)

Additionally, the pH levels prior to implantation were significantly different, with a median pH of 7.19 for survivors and 7.10 for non-survivors (*p* < 0.001), see Supplemental Fig. 3. Regression analysis indicated a strong and significant influence of pH on survival (Fig. 4B, *p* < 0.001. For ECPR and shock patients, pH levels prior to implantation were significantly higher in survivors compared to non-survivors (both *p* < 0.001, see Fig. 4, Tables 2 and 3).

**Table 2:** Hospital survivors and non-survivors (ECPR)

Variable	*N*	Survived *N* = 80	Non-survived *N* = 204	*p* value
Age, median (IQR)	284	60 (49, 64)	61 (51, 69)	0.103
Male gender, * n* (%)	284	59 (74%)	162 (79%)	0.430
Witnessed arrest, * n* (%)	281	75 (96%)	183 (90%)	0.107
No-flow duration (minutes), median (IQR)	206	0 (0, 1)	0 (0, 4)	0.261
Bystander CPR, * n* (%)	257	48 (73%)	138 (72%)	0.989
Mechanical CPR, * n* (%)	264	39 (52%)	141 (75%)	< 0.001
Low-flow duration (minutes), median (IQR)	259	34 (20, 54)	55 (30, 72)	< 0.001
Lactate pre-implant (mmol/l), median (IQR)	252	9.0 (5.4, 14.0)	12.4 (9.0, 16.0)	< 0.001
pH pre-implant, median (IQR)	252	7.11 (7.02, 7.21)	7.00 (6.90, 7.13)	< 0.001
Location of ECPR, * n* (%)	282			0.104
IHCA		45 (57%)	92 (45%)	
OHCA		34 (43%)	111 (55%)	
Venting, * n* (%)	284	18 (23%)	41 (20%)	0.848
Time on ICU, median (IQR)	255	20 (12, 27)	2 (1, 5)	< 0.001
Successful ECMO weaning, * n* (%)	284	77 (97%)	30 (15%)	< 0.001
30-day survival, * n* (%)	283	79 (100%)	8 (3.9%)	< 0.001
Hospital survival, * n* (%^$^)	284	80 (28%)	204 (72%)	
CPC at discharge, * n* (%)	283			< 0.001
CPC 1–2		66 (84%)	0 (0%)	
CPC 3–4		13 (16%)	0 (0%)	
CPC 5		0 (0%)	204 (100%)	

Characteristics of patients in the ECPR subgroup divided by hospital survivors and non-survivors. The column 'N' denotes the overall sample size with available values for each variable, irrespective of group; percentages reflect solely the observed data without any imputation. § of whole subgroup with ECPR

*CPC* cerebral performance category, *CPR* cardiopulmonary resuscitation, *ECMO* extracorporeal membrane oxygenation, *ECPR* extracorporeal cardiopulmonary resuscitation, *ICU* intensive care unit, *IHCA* in-hospital cardiac arrest, *IQR* interquartile range, *OHCA* out-of-hospital cardiac arrest

**Table 3:** Hospital survivors and non-survivors (shock)

Variable	*N*	Survived *N* = 112	Non-survived *N* = 90	*p* value
Age, median (IQR)	202	59 (52, 66)	65 (54, 71)	< 0.001
Male gender, * n* (%)	202	80 (71%)	64 (71%)	0.968
Type of shock, * n* (%)	202			0.412
Cardiogenic shock		90 (80%)	76 (84%)	
Non-cardiogenic shock		22 (20%)	14 (16%)	
Lactate pre-implant (mmol/l), median (IQR)	181	5.3 (3.2, 8.9)	9.0 (5.6, 13.1)	< 0.001
pH pre-implant, median (IQR)	175	7.25 (7.13, 7.36)	7.20 (7.10, 7.35)	0.109
Venting, * n* (%)	202	34 (30%)	18 (20%)	0.152
Time on ICU, median (IQR)	192	20 (11, 31)	9 (2, 18)	< 0.001
Successful ECMO weaning, * n* (%)	202	109 (98%)	20 (22%)	< 0.001
30-day survival, * n* (%)	199	101 (94%)	3 (3.3%)	< 0.001
Hospital survial, * n* (%^§^)	202	112 (55%)	90 (45%)	
CPC at discharge, * n* (%)	201			< 0.001
CPC 1–2		92 (83%)	0 (0%)	
CPC 3–4		19 (17%)	0 (0%)	
CPC 5		0 (0%)	90 (100%)	

Characteristics of patients in the ECPR subgroup divided by hospital survivors and non-survivors. The column '*N*' denotes the overall sample size with available values for each variable, irrespective of group; percentages reflect solely the observed data without any imputation. § of whole subgroup with shock

*CPC* cerebral performance category, *ECMO* extracorporeal membrane oxygenation, *ICU* intensive care unit, *IQR* interquartile range

In a multivariate logistic regression analysis of hospital survival in all patients, all three investigated parameters evaluated here (age, lactate, and pH, *p* < 0.001, respectively) were independent predictors of survival (see, Supplemental Material 1).

## Discussion

The establishment of VERGE, the first national VA ECMO registry in Germany provides a unique perspective on the implementation and outcomes of this life-sustaining technology. This first report highlights several important aspects of routine VA ECMO support.

### Survival

Primary endpoint of this report was hospital survival—which varied significantly with the indication for ECMO. While survival was 58% in shock and 28% in ECRP. These are well in the range of the ELSO-report, stating 46% and 31%, respectively [8]. Data of randomized trials in VA ECMO in acute myocardial infarction associated shock show a similar 30-day survival of 52% [9]. Randomized data on ECPR had a comparable survival in the ECPR arm (32% CPC1-2 180-day survival [10] and 20% CPC 1–2 30-day survival [11]. These survival rated, however, cannot be directly compared as only CPC 1–2 survival was measured, follow-up was longer and not all patients revived an ECPR in this arm. In VERGE, CPC 1–2 at discharge was 23%. The poor survival rate of the peri-procedural group is alarming. As the number of patients in this subgroup is still extremely low, no further conclusions can be drawn. Other studies to peri-procedural ECMO show better results [12, 13].

### Treatment

Compared to randomized studies, a relatively high proportion of LV venting (23%) was observed. For instance, patients included in the ECLS-SHOCK study were treated with a venting device in 5.8% of the cases [9]. In the first year of the registry's existence, it is too early to draw conclusions from this observation.

The length of intensive care unit (ICU) stay differed significantly between survivors and non-survivors. For patients in cardiogenic shock, the ICU stay was 20 days versus 9 days (*p* < 0.001), and for the cohort of eCPR patients, it was 20 days versus 2 days (*p* < 0.001). Given the resource-intensive nature of these therapies, the duration of such treatments must be carefully considered before initiating an ECMO program.

### Age

The median age at cannulation was 61 years for patients undergoing VA ECMO for shock and 60 years for ECPR, underscoring a trend toward the higher utilization in older patients. This reflects a broader shift within the age spectrum and supports the well-documented correlation between increased age and reduced survival likelihood [14, 15]. Our findings corroborate the relationship between survival rates and age, yet they do not pinpoint a clear cutoff age that significantly alters outcomes, moreover, in our data correlation was weak. This absence of a definitive age threshold poses critical questions about the other potential factors influencing outcomes, which might be deduced from this comprehensive dataset. The fact that patients over the age of 80 are being treated with ECPR epitomizes the challenges faced by medical teams. These challenges are not solely clinical but encompass the timely and effective use of VA ECMO before complete patient information is available.

### Biomarkers

The lactate levels in surviving shock patients had a median of 5.3 mmol/L, similar to those of the patients enrolled in the ECLS Shock trial [9], while non-survivors exhibited significantly higher levels. A correlation of prognosis and lactate has been shown earlier [16]. Similarly, in ECPR patients, lactate levels were significantly different between survivors and non-survivors. Logistic regression analysis demonstrated a substantial influence of lactate levels on survival probability. It is not known how many centers are capable of measuring lactate levels above 20 or 25 mmol/L. This may have affected the categorization into lactate groups. However, in VERGE, no patient with a lactate above 25 mmol/L survived, which might be a cornerstone for Indication for VA ECMO.

Further conclusions may be drawn from aggregated data in subsequent years. Moreover, the pH was a strong predictor of survival in both ECPR as well as shock. Importantly, pH itself is an aggregate of respiratory and metabolic acid–base disturbances and, therefore, linked to lactate. With data shown here, we cannot differentiate between respiratory or metabolic acidosis. In VERGE, no patient with a pH below 6.7 survived and prognosis was best in patients with an alkalosis. These registry data, however, cannot be used to justify a hyperventilation strategy of VA ECMO patients and further research is needed.

### Limitations

The reported data are subject to a reporting bias. The 14 contributing centers all have a high level of ECMO activity and a stronger inclination toward scientific research, meaning that the report does not yet reflect the true state of patient care across Germany. With the annual publication of the registry, the number of participating centers is expected to increase. The aim of this registry is to provide a comprehensive description of the VA ECMO landscape in Germany. A concise data structure is essential to ensure high adherence to the registry, which may result in some imprecise definitions. For instance, unlike in prospective randomized studies, the definition of cardiogenic shock was left to the discretion of the participating centers.

## Conclusion

VERGE is the first national VA ECMO registry in Germany. Survival in the registry is similar to randomized data and the ELSO registry. Hospital survival independently correlated with age, lactate, and pH.

## Supplementary Information

Below is the link to the electronic supplementary material.

Supplementary file1 (JPG 81 KB)

Supplementary file2 (JPG 89 KB)

Supplementary file3 (JPG 82 KB)

Supplementary file4 (DOCX 14 KB)

## Data Availability

The data underlying this study are available from the corresponding author upon reasonable request.

## References

[1] Paddock S, Meng J, Johnson N, Chattopadhyay R, Tsampasian V, Vassiliou V (2024) The impact of extracorporeal membrane oxygenation on mortality in patients with cardiogenic shock post-acute myocardial infarction: a systematic review and meta-analysis. Eur Hear J Open 4:3. 10.1093/ehjopen/oeae003 10.1093/ehjopen/oeae003 PMC10836884 38313078

[2] Albulushi A, Tawfek A, Lawatia HA (2024) Evaluating the efficacy and safety of temporary mechanical circulatory support devices in acute cardiogenic shock: a subgroup-specific systematic review. Curr Probl Cardiol 49:102619. 10.1016/j.cpcardiol.2024.102619 38723794 10.1016/j.cpcardiol.2024.102619

[3] Lang CN, Kaier K, Zotzmann V, Stachon P, Pottgiesser T, Muehlen C et al (2021) Cardiogenic shock: incidence, survival and mechanical circulatory support usage 2007–2017-insights from a national registry. Clin Res Cardiol 110:1421–1430. 10.1007/s00392-020-01781-z 33258007 10.1007/s00392-020-01781-z PMC8405485

[4] Harris PA, Taylor R, Thielke R, Payne J, Gonzalez N, Conde JG (2009) Research electronic data capture (REDCap)—A metadata-driven methodology and workflow process for providing translational research informatics support. J Biomed Inform 42:377–381. 10.1016/j.jbi.2008.08.010 18929686 10.1016/j.jbi.2008.08.010 PMC2700030

[5] Harris PA, Taylor R, Minor BL, Elliott V, Fernandez M, O’Neal L et al (2019) The REDCap consortium: building an international community of software platform partners. J Biomed Inform 95:103208. 10.1016/j.jbi.2019.103208 31078660 10.1016/j.jbi.2019.103208 PMC7254481

[6] Team RC (2021) R: a language and environment for statistical computing. Austria, Vienna

[7] Wickham H (2009) ggplot2, elegant graphics for data. Analysis. 10.1007/978-0-387-98141-3

[8] ECLS International Summary of Statistics n.d. https://www.elso.org/registry/internationalsummaryandreports/internationalsummary.aspx (accessed July 3, 2024).

[9] Thiele H, Zeymer U, Akin I, Behnes M, Rassaf T, Mahabadi AA et al (2023) Extracorporeal life support in infarct-related cardiogenic shock. N Engl J Med. 10.1056/nejmoa2307227 37634145 10.1056/NEJMoa2307227

[10] Belohlavek J, Smalcova J, Rob D, Franek O, Smid O, Pokorna M et al (2022) Effect of intra-arrest transport, extracorporeal cardiopulmonary resuscitation, and immediate invasive assessment and treatment on functional neurologic outcome in refractory out-of-hospital cardiac arrest. JAMA 327:737–747. 10.1001/jama.2022.1025 35191923 10.1001/jama.2022.1025 PMC8864504

[11] Suverein MM, Delnoij TSR, Lorusso R, Bruinsma GJBB, Otterspoor L, Kraemer CVE et al (2023) Early extracorporeal CPR for refractory out-of-hospital cardiac arrest. N Engl J Med 388:299–309. 10.1056/nejmoa2204511 36720132 10.1056/NEJMoa2204511

[12] van den Buijs DMF, Wilgenhof A, Knaapen P, Zivelonghi C, Meijers T, Vermeersch P et al (2022) Prophylactic impella CP versus VA-ECMO in patients undergoing complex high-risk indicated PCI. J Interv Cardiol 2022:8167011. 10.1155/2022/8167011 36447936 10.1155/2022/8167011 PMC9663242

[13] Bulnes JF, Martínez A, Sepúlveda P, Fuensalida A, Besa S, Garrido L et al (2024) Outcomes of a modified, low-cost, veno-arterial extracorporeal membrane oxygenation (V-A ECMO) for elective, periprocedural support of high-risk percutaneous cardiac interventions: an experience from a latinamerican center. Perfusion 39:998–1005. 10.1177/02676591231178413 37226290 10.1177/02676591231178413

[14] Fernando SM, MacLaren G, Barbaro RP, Mathew R, Munshi L, Madahar P et al (2023) Age and associated outcomes among patients receiving venoarterial extracorporeal membrane oxygenation–analysis of the Extracorporeal Life Support Organization registry. Intensiv Care Med 8:1–11. 10.1007/s00134-023-07199-1 10.1007/s00134-023-07199-1 37792052

[15] Friedrichson B, Mutlak H, Zacharowski K, Piekarski F (2021) Insight into ECMO, mortality and ARDS: a nationwide analysis of 45,647 ECMO runs. Crit Care 25:38. 10.1186/s13054-021-03463-2 33509228 10.1186/s13054-021-03463-2 PMC7841040

[16] Fuernau G, Desch S, de Waha-Thiele S, Eitel I, Neumann F-J, Hennersdorf M et al (2020) Arterial lactate in cardiogenic shock: prognostic value of clearance versus single values. JACC Cardiovasc Interv 13:2208–2216 33032708 10.1016/j.jcin.2020.06.037

