# Supplementary figures and images for: Clinical use and predictors of outcome in venoarterial extracorporeal membrane (VA ECMO): insights from VERGE (VA ECMO Registry of Germany)
Source: Clin Res Cardiol. 2025 Apr 22;114(10):1377–87. doi: 10.1007/s00392-025-02650-3 (PMC12460512; doi:10.1007/s00392-025-02650-3)

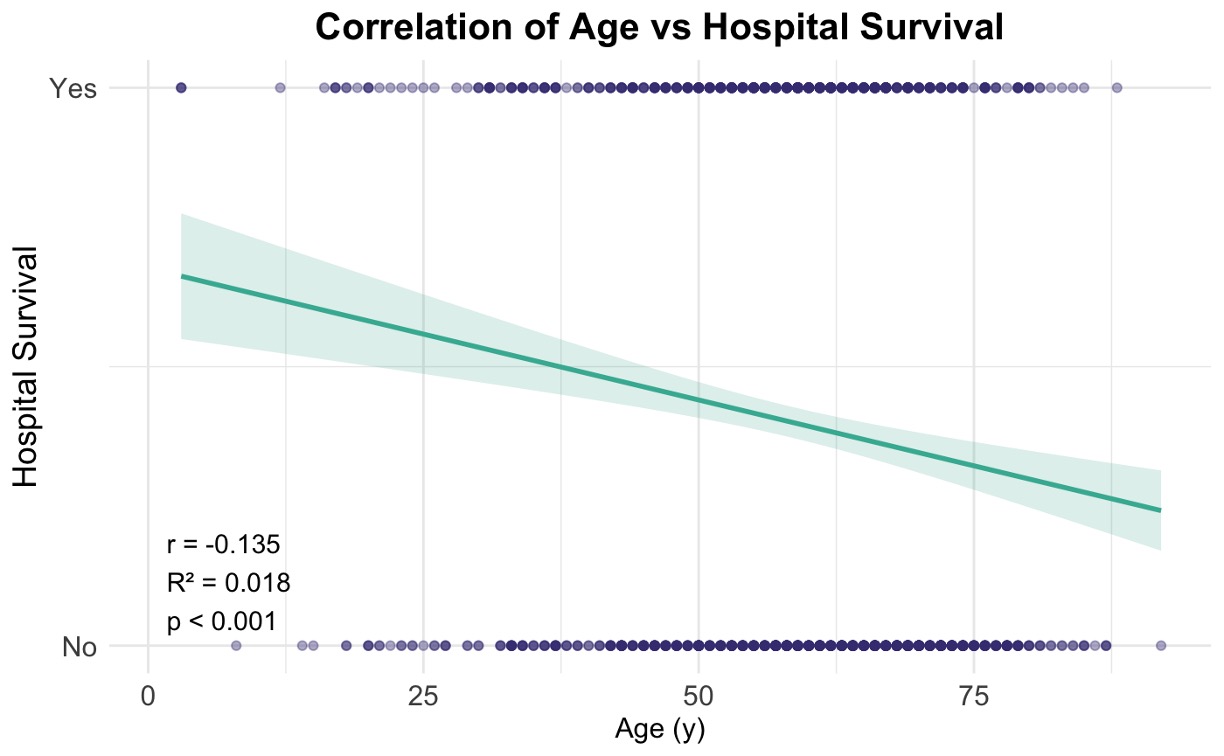

Supplement: Supplementary file 1 — Supplementary file1 (JPG 81 KB) [file 392_2025_2650_MOESM1_ESM.jpg]

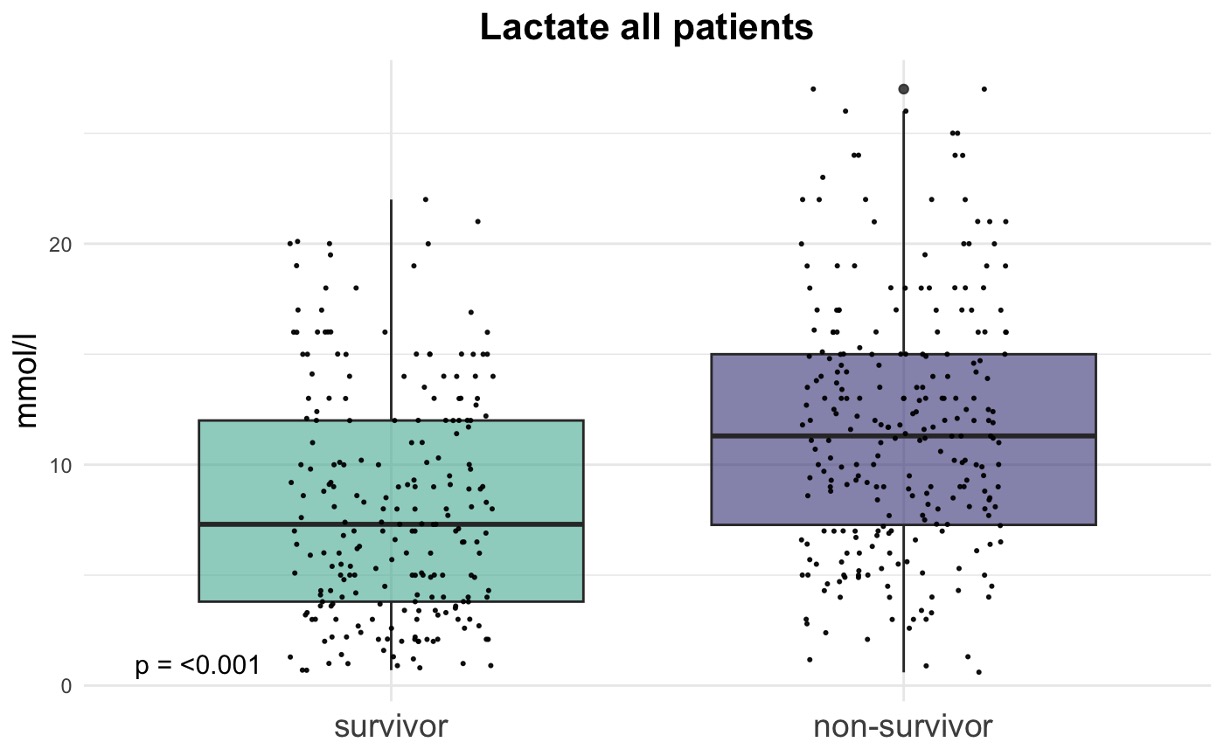

Supplement: Supplementary file 2 — Supplementary file2 (JPG 89 KB) [file 392_2025_2650_MOESM2_ESM.jpg]

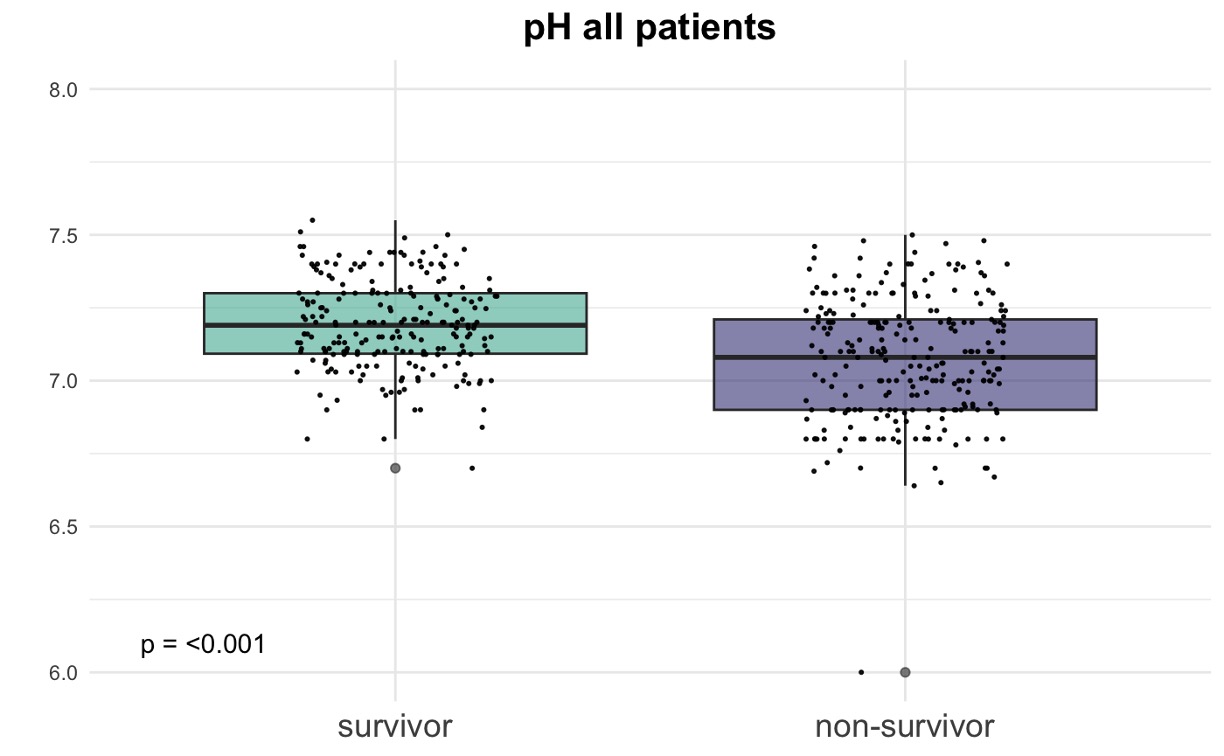

Supplement: Supplementary file 3 — Supplementary file3 (JPG 82 KB) [file 392_2025_2650_MOESM3_ESM.jpg]
