# Supplementary material for: Clinical use and predictors of outcome in venoarterial extracorporeal membrane (VA ECMO): insights from VERGE (VA ECMO Registry of Germany)
Source: Clin Res Cardiol. 2025 Apr 22;114(10):1377–87. doi: 10.1007/s00392-025-02650-3 (PMC12460512; doi:10.1007/s00392-025-02650-3)
Supplement: Supplementary file 4 — Supplementary file4 (DOCX 14 KB) [file 392_2025_2650_MOESM4_ESM.docx]

| **Variable** | **Odds Ratio** | **95% Confidence Interval** | ***p-*value** |
| --- | --- | --- | --- |
| Age (per 10y) | 0.721 | 0.618-0.837 | <0.0001 |
| Lactate (per mmol/l) | 0.913 | 0.874-0.952 | <0.0001 |
| pH (per 0.1) | 1.234 | 1.091-1.400 | 0.0001 |

Supplemental table 1: multivariate logistic regression for hospital survival of all patients in registry
